# Sensitivity and specificity of DPP^®^ Fever Panel II Asia in the diagnosis of malaria, dengue and melioidosis

**DOI:** 10.1099/jmm.0.001584

**Published:** 2022-08

**Authors:** Premjit Amornchai, Viriya Hantrakun, Gumphol Wongsuvan, Chaiyaporn Boonsri, Sasinaphon Yoosuk, Jiraporn Nilsakul, Stuart D Blacksell, T Eoin West, Yoel Lubell, Direk Limmathurotsakul

**Affiliations:** 1 Mahidol-Oxford Tropical Medicine Research Unit, Faculty of Tropical Medicine, Mahidol University, Bangkok, Thailand; 2 Medical Department, Sunpasitthiprasong Hospital, Ubon Ratchathani, Thailand; 3 Pathology Department, Sunpasitthiprasong Hospital, Ubon Ratchathani, Thailand; 4 Centre for Tropical Medicine and Global Health, Nuffield Department of Medicine, University of Oxford, United Kingdom; 5 Division of Pulmonary and Critical Care Medicine, Harborview Medical Center, University of Washington, Seattle, Washington, United States of America; 6 Department of Microbiology and Immunology, Faculty of Tropical Medicine, Mahidol University, Bangkok, Thailand; 7 Department of Tropical Hygiene, Faculty of Tropical Medicine, Mahidol University, Bangkok, Thailand

**Keywords:** Rapid diagnostic test, DPP Fever Panel II Asia, accuracy, sensitivity, specificity, melioidosis, dengue, malaria, chikungunya, zika virus infection

## Abstract

**Introduction:**

Rapid diagnostic tests (RDTs) that can facilitate the diagnosis of a panel of tropical infectious diseases are critically needed. The ‘DPP^®^ Fever Panel II Asia’ is a multiplex lateral flow immunoassay comprising of antigen and IgM panels for the diagnosis of pathogens that commonly cause febrile illness in Southeast Asia.

**Aim:**

To evaluate sensitivity and specificity of DPP^®^ Fever Panel II for malaria, dengue and melioidosis.

**Methodology:**

We conducted a cohort-based case-control study. Both cases and controls were derived from a prospective observational study of patients presenting with community-acquired infections and sepsis in northeast Thailand (Ubon-sepsis). We included 143 and 98 patients diagnosed with malaria or dengue based on a positive PCR assay and 177 patients with melioidosis based on a culture positive for *Burkholderia pseudomallei*. Controls included 200 patients who were blood culture positive for *Staphylococcus aureus*, *Escherichia coli* or *Klebsiella pneumoniae*, and cases of the other diseases. Serum samples collected from all patients within 24 hours of admission were stored and tested using the DPP^®^ Fever Panel II Asia antigen and IgM multiplex assays. We selected cutoff values for each individual assay corresponding to a specificity of ≥95%. When assessing diagnostic tests in combination, results were considered positive if either individual test was positive.

**Results:**

Within the DPP^®^ Fever Panel II antigen assay, a combination of pLDH and HRPII for malaria had a sensitivity of 91% and a specificity of 97%. The combination of dengue NS1 antigen and dengue antibody tests had a sensitivity of 61% and a specificity of 91%. The *B. pseudomallei* CPS antigen test had a sensitivity of 27% and a specificity of 97%. An odds ratio of 2.34 (95% CI 1.16-4.72, p=0.02) was observed for the association between positivity of CPS and mortality among melioidosis patients.

**Conclusion:**

The performance of the DPP^®^ Fever Panel II Asia for diagnosis of malaria was high and that for dengue and melioidosis was relatively limited. For all three diseases, performance was comparable with other established RDTs. The potential operational advantages of a multiplex and quantitative point of care assay are substantial and warrant further investigation.

## Introduction

Malaria, dengue and melioidosis are common tropical infectious diseases in Southeast Asia [1, 2]. In 2020, the World Health Organization (WHO) reported 229 million malaria cases worldwide and 409,000 deaths [3]. A modelling study estimated 104 million dengue cases per year worldwide and 40,467 deaths [4]. For Melioidosis (an infectious disease caused by the Gram-negative bacterium *Burkholderia pseudomallei* [2]), a modelling study estimated 165,000 cases per year worldwide and 89,000 deaths [5]. Malaria [6], dengue [7] and melioidosis patients [8] commonly present as an acute febrile illness or sepsis, a syndrome defined by a dysregulated host response to infection resulting in significant organ dysfunction and death that can be caused by a variety of agents, including bacteria, fungi, and viruses [9].

Clinically, differentiating between these and other common tropical diseases can be challenging. Over the last decade the use of rapid diagnostics tests (RDTs) to detect the presence of malaria parasites has become routine and in 2020 almost half a billion malaria RDTs were procured for use across the endemic world [3]. Singleplex, qualitative RDTs are increasingly available for a wide array of tropical diseases that might aid in the diagnosis of other non-malarial diseases; however, there are clear logistical and operational challenges to implementing numerous RDTs for a broad range of common pathogens at both the health care system and patient management levels.

Another potential drawback of qualitative RDTs is a fixed, built-in cutoff value the selection of which will trade-off sensitivity against specificity. The justification for these thresholds is not always clear and in many settings could be sub-optimal. While the need for high sensitivity is intuitively clear, RDTs with low specificity can have limited utility for clinical and policy decision making in many endemic settings. For example, indirect hemagglutination assays (IHA) is the most widely used serological assay for exposure to *B. pseudomallei* [10]. In a recent study in Thailand, IHA had a sensitivity of 69.5% and a specificity of 67.6% due to high background seropositivity [11]. Use of such a low specificity test in endemic settings can imply high levels of overdiagnoses and overuse of high cost, last line antibiotics effective against *B. pseudomallei*. This places patients at risk of adverse drug reactions and further catalyzes the spread of antimicrobial resistance. It can also skew mortality estimates by inflating the overall number of cases and underestimating case fatality rates, misguiding the public health response to melioidosis in endemic areas [12]. RDTs for malaria with high sensitivity and low specificity can pose similar problems, particularly in high transmission areas, resulting in high false positive rates, unnecessary anti-malarial treatment and undertreatment of bacterial infections [13-15].

Multiplex, quantitative RDTs that can diagnose a panel of common tropical infectious diseases with high specificity among patients presenting with acute febrile illness or sepsis in the tropics could offer substantial advantages over the use of singleplex qualitative RDTs. First and foremost, they could aid in the diagnosis of multiple diseases with similar clinical presentations simultaneously. Second, is the potential use of population specific cutoffs to achieve particular objectives, such as maintaining sufficiently high specificity to ensure clinical utility. Finally, at the patient level a quantitative readout for some antigens could also act as markers of severity, as has been shown for HRPII in malaria [16], for CPS in melioidosis [17] and in some studies for NS1 in dengue [18].

Chembio Diagnostic Inc, in collaboration with FIND (Foundation for Innovative New Diagnostics), developed a multiplex lateral flow immunoassay (DPP^®^ Fever Panel II Assay) that is able to detect serum immunoglobulin M (IgM) and specific microbial antigen of the most common agents of acute febrile illness (AFI) in Asia. Here, we evaluated the DPP^®^ Fever Panel II Asia Antigen System and DPP^®^ Fever Panel II Asia IgM System for diagnosis of malaria, dengue and melioidosis.

## Methods

We previously conducted a prospective observational (non-interventional) study of community-acquired infection and sepsis in Sunpasitthiprasong Hospital, Ubon Ratchathani province, northeast Thailand [8]. From March 2013 to February 2017, we enrolled 5,001 adult patients (≥18 years of age) who were admitted with a primary diagnosis of suspected or documented infections as determined by the attending physician, were within 24 h of hospital admission and had at least three sepsis diagnostic criteria documented in their medical records. We excluded patients who were suspected of having hospital-acquired infections determined by the attending physician, had a hospital stay within 30 days prior to this admission or were transferred from another hospital with a total duration of hospitalization >72 hours. Blood was drawn from all patients at the time of enrolment for culture and polymerase chain reaction (PCR). Patients who were positive for malaria, dengue or melioidosis were selected as cases in the current study. Dengue and malaria were diagnosed by a nested PCR assay as described previously [6, 7, 19]. Patients who were culture positive for *B. pseudomallei* from any clinical specimens were selected as cases of melioidosis. Patients with blood cultures positive for *Staphylococcus aureus*, *Escherichia coli* or *Klebsiella pneumoniae*, or cases of the other diseases were selected as controls.

**Figure 1:**
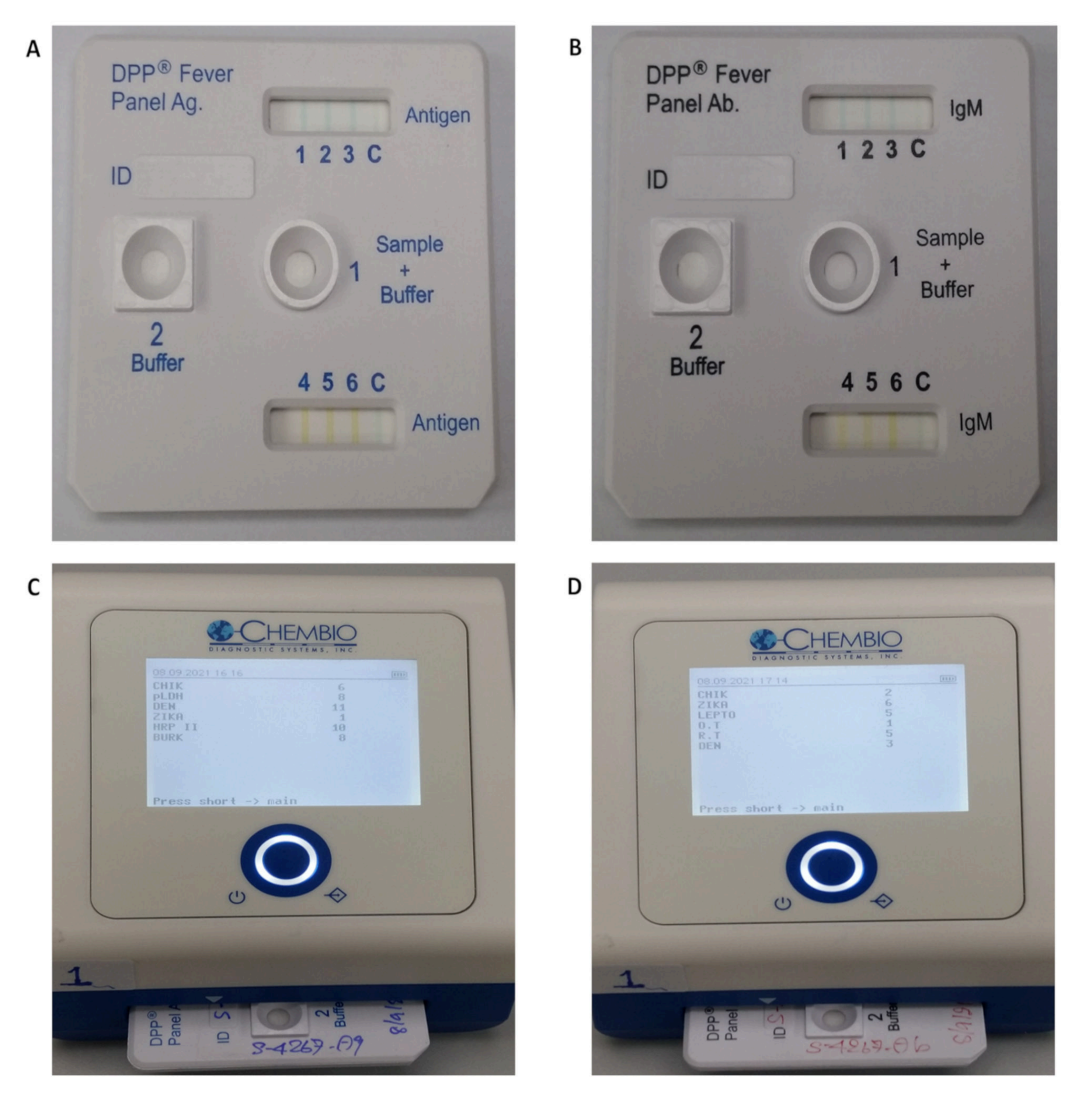
DPP^®^ Fever Panel II Asia Antigen System and IgM System. (A) DPP^®^ Fever Panel II Asia Antigen System, (B) DPP^®^ Fever Panel II Asia IgM System, (C) Example test result of the antigen system read by DPP^®^ Micro Reader 2, and (D) Example test result of the IgM system read by DPP^®^ Micro Reader 2.

**Figure 2:**
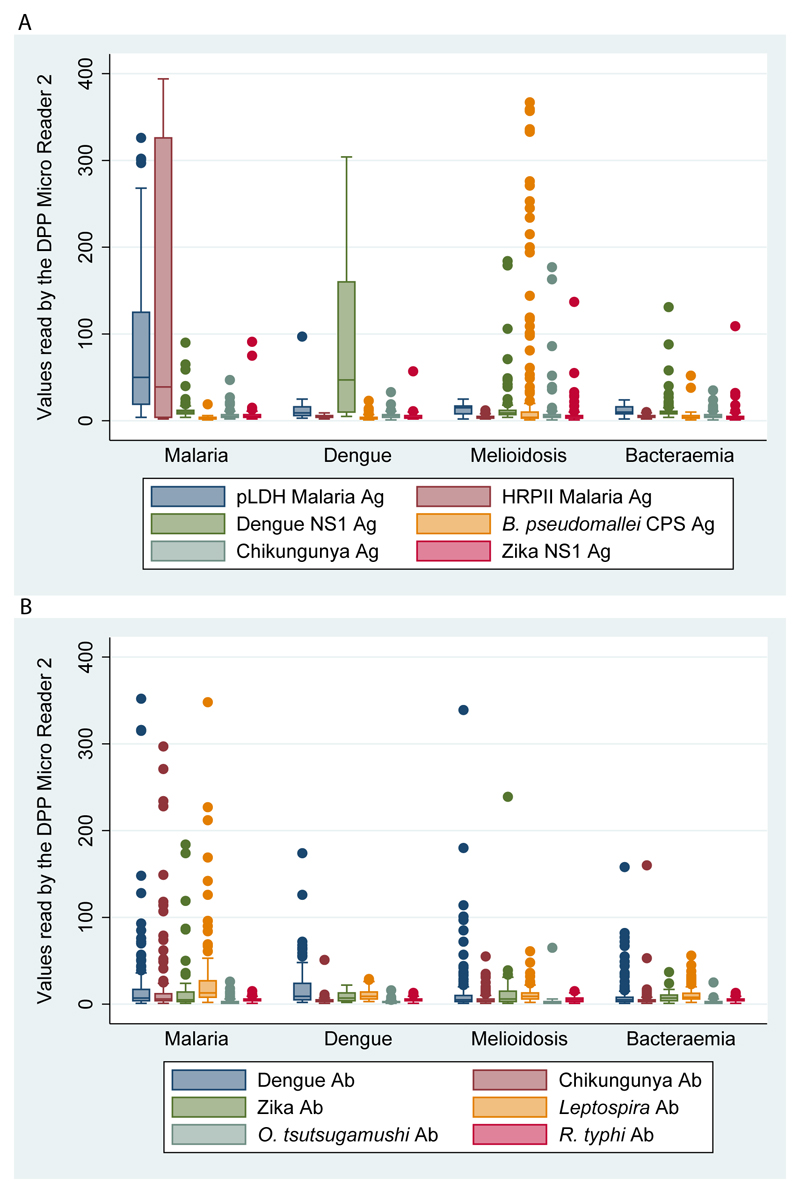
Quantitative test results for antigen-detection (A) and IgM-detection (B) by DPP^®^ Fever Panel II Asia Antigen System and IgM System among patients with malaria, dengue, melioidosis and bacteraemia. Boxes show 25th, 50th, and 75th percentiles. Bottom and top whiskers show 25th percentile minus 1.5 times the interquartile range (IQR) and 75th percentile plus 1.5 times the IQR, respectively. Patients with bacteraemia included patients with blood culture positive for *E. coli*, *K. pneumoniae* and *S. aureus*. The three patients with mixed infection between melioidosis and dengue are shown in both groups; the patient with mixed infection between malaria and dengue is shown in both groups; and the patient with mixed infection between melioidosis and *K. pneumoniae* bacteraemia is shown only in the melioidosis group.

Serum samples obtained in the first 24 hours of admission were frozen at -80°C and for the purposes of the current study thawed and tested using the DPP^®^ Fever Panel II Asia antigen and IgM multiplex assays. The DPP^®^ Fever Panel II Asia **antigen** assay (Chembio Inc; lot no FPIIAAG010821/A; Figure 1A) is a multi-line lateral flow for the simultaneous qualitative detection and differentiation of specific febrile illness antigens; including (1) Chikungunya, (2) pan *Plasmodium* antigen lactate dehydrogenase (pLDH), (3) Dengue NS1, (4) Zika NS1, (5) *Plasmodium falciparum* (Pf) Histidine rich protein II antigen (HRPII) and (6) *Burkholderia* Capsular Polysaccharide antigen (CPS). The DPP^®^ Fever Panel II Asia **IgM** assay (Chembio Inc; lot no FPIIAAB010821/A; Figure 1B) was developed for the simultaneous qualitative detection and differentiation of specific IgM antibodies for (1) Chikungunya, (2) Zika, (3) *Leptospira*, (4) *Orientia tsutsugamushi*, (5) *Rickettsia typhi* and (6) Dengue. The DPP^®^ Fever Panel II Asia antigen and IgM multiplex assays are not yet commercially available, and cutoff values to operationalize their quantitative readouts are not yet finalized. The tests were performed as described by the manufacturer, using preliminary cutoffs for interpretation, calibrated to meet a specificity of ~95% for each of the individual targets. For the antigen assay, 50 μL of serum sample was directly added in the sample well and following immediately added by 100 μL (4 drops) of sample buffer into the SAMPLE + BUFFER well (Well #1; Figure 1A). For the IgM assay, 10 μL of serum sample was mixed with 150 μL of sample buffer in separate tube and used for 100 μL to add into the SAMPLE + BUFFER well (Well #1; Figure 2A). At 5 minutes of incubation at room temperature for both systems, 300 μL (12 drops) of running buffer was added into the BUFFER well (Well #2). The test results were interpreted using the DPP^®^ Micro Reader 2 between 20 and 25 minutes after buffer addition to Well #1 (Figure 1C and 1D). Tests were labelled with the patient code and performing date. The laboratory technicians were unaware of the final diagnosis when performing the tests.

The lowest OD cutoff values that gave a specificity of ≥95% for each individual test line were selected. Sensitivity was defined as the proportion of case patients who had positive test results. Specificity was defined as the proportion of control patients who had negative test results. A receiver operating characteristic (ROC) curve was created to monitor the shifting of the positive cutoff value of true-positive (sensitivity) and false positive (1-specificity) rates. When assessing diagnostic tests in combination, results were considered positive if either test was positive. A secondary analysis was also conducted using the Youden index (sensitivity + specificity – 1) to identify a cutoff with the highest overall sensitivity and specificity. We also used logistic regression models to assess the association between CPS as measured on the DPP^®^ Antigen assay and mortality among patients with melioidosis, of whom 94/177 (53%) died. A similar analysis for HRPII in malaria patients, and NS1 in dengue patients was not possible due to the low number of deaths in these cases (1/153 and 4/126 respectively).

All analyses were performed using Stata version 14 (Stata Corp LP, College Station, TX, USA). The final database with data dictionary are publicly available online (doi:10.6084/m9.figshare.19029977).

## Results

From March 2013 to February 2017, 5,001 adult patients presenting with community-acquired infections or sepsis were enrolled and followed for 28 days. A total of 153 and 126 were PCR positive for malaria or dengue, and were included as malaria and dengue cases, respectively. A total of 193 patients were culture positive for *B. pseudomallei* and thus included as melioidosis cases, and 268 patients who were blood culture positive for *E. coli* (n=189), *K. pneumoniae* (n=26) and *S. aureus* (n=53) without malaria, dengue and melioidosis were included in this study as bacteraemia controls. Serum was not available for all patients. Overall, 614 patients were included in the analyses; including 143 malaria cases (70 Pf, 63 *Plasmodium vivax* (Pv) and 10 mixed Pf and Pv), 98 dengue cases, 177 melioidosis cases and 200 patients with bacteraemia were included in the analyses. Of 414 cases of malaria, dengue or melioidosis, 5 had mixed infections including melioidosis plus dengue (n=3), Pv malaria plus dengue (n=1) and melioidosis plus *K. pneumoniae* bacteraemia (n=1).

The baseline characteristics of patients included in the analysis are described in Table S1. The proportion of male was highest among malaria patients (90%; 128/143) and lowest among dengue patients (44%; 43/98). The median age was lowest among dengue patients (27 years, range 18 to 75 years) and highest among bacteraemia patients (64 years, range 18 to 94 years). Of 143 malaria, 98 dengue and 177 melioidosis patients, 92 (64%), 68 (69%) and 95 (54%) had a duration of symptoms less than 5 days prior to hospitalization.

**Figure 3:**
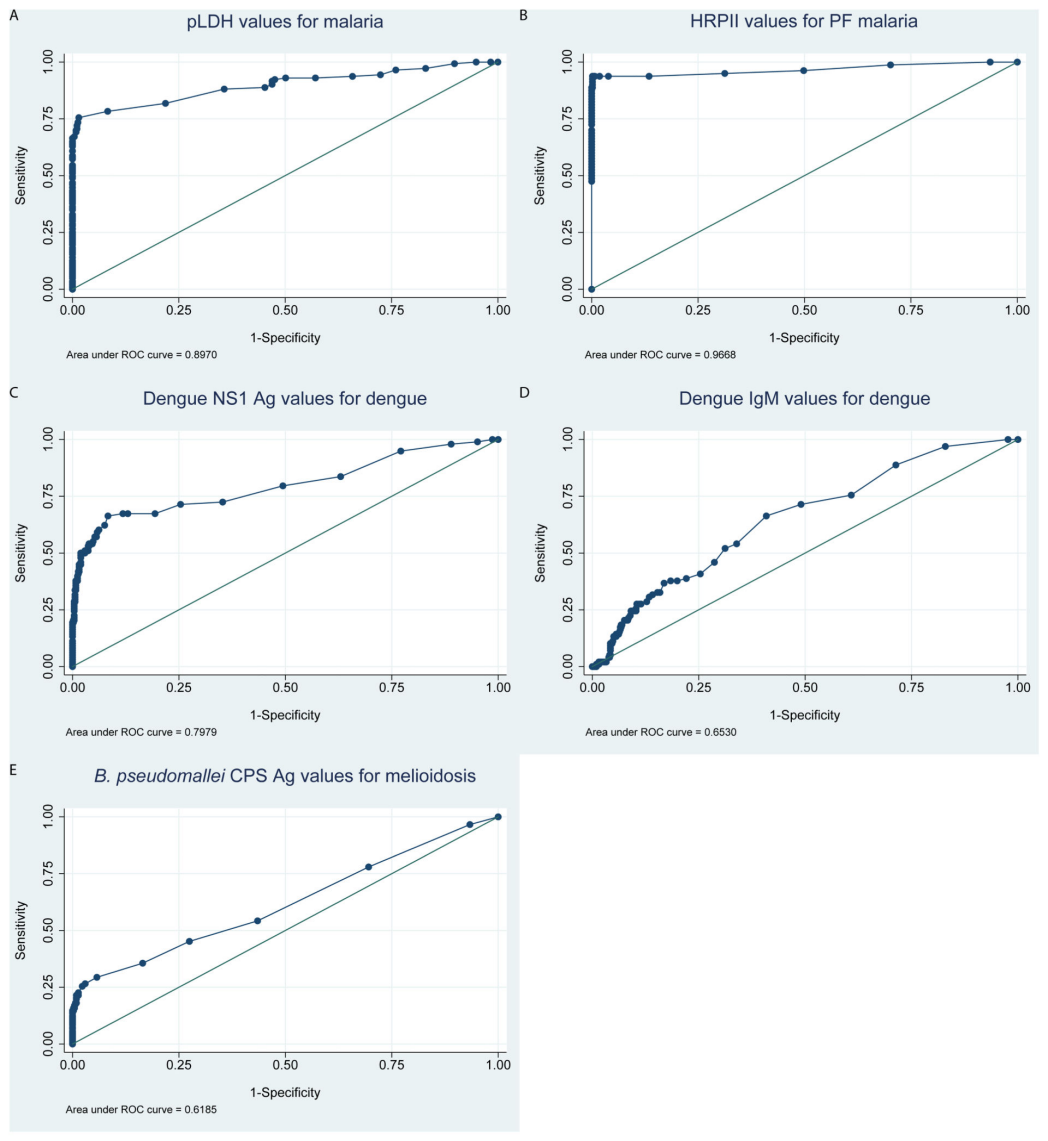
Receiver operating characteristic curves of DPP^®^ Fever Panel II Asia Antigen System and IgM System for diagnosis of malaria, dengue and melioidosis.

Figure 2A and 2B show quantitative values of antigen-detection and IgM-detection read by the DPP^®^ Fever Panel II Asia antigen and IgM assays, respectively. For example, the median value of pLDH among malaria cases was 50 (IQR 19-125; range 4-326). Areas under the ROC curves (AUROCC) of pLDH for malaria diagnosis were 0.90 (95%CI 0.86 to 0.93; Figure 3A) and AUROCC of HRPII malaria antigen test for Pf-malaria diagnosis was 0.97 (95% 0.94-1.00; Figure 3B). AUROCC of Dengue NS1 Ag test and Ab test for dengue diagnosis were 0.80 (95% 0.74-0.86; Figure 3C) and 0.65 (95%CI 0.60-0.71; Figure 3D), respectively. AUROCC of *Burkholderia* CPS Ag for melioidosis diagnosis was 0.62 (95%CI 0.57-0.67; Figure 3E).

Using a positive cutoff value of 19 for pLDH malaria antigen test, the test had a sensitivity of 76% (108/143 malaria case patients) and a specificity of 99% (464/471 non-malaria control patients; Table 1). Of 80 and 63 patients with Pf and Pv mono infections, pLDH malaria antigen test had a sensitivity of 73% (58/80 Pf case patients) and 79% (50/63 Pv-mono malaria case patients). Using a positive cutoff value of 9 for HRPII malaria antigen test, the test had a sensitivity of 94% (75/80 Pf malaria case patients) and a specificity of 98% (524/534 non-Pf malaria control patients). Among 10 non-Pf malaria control patients who had HRPII malaria antigen test ≥9, 2 had Pv malaria, 2 were dengue cases, 3 were melioidosis cases and 3 were *K. pneumoniae* bacteraemia cases. The combination of pLDH and HRPII malaria antigen test had a sensitivity of 91% (130/143 malaria case patients) and a specificity of 97% (458/471 non-malaria control patients).

Using a positive cutoff value of 22 for Dengue NS1 Antigen test, the test had a sensitivity of 55% (54/98 dengue case patients) and a specificity of 95% (491/516 non-dengue control patients). Using a positive cutoff value of 56 for dengue antibody test, the test had a sensitivity of 11% (11/98 dengue case patients) and a specificity of 95% (491/516 non-dengue control patients) for dengue. Of 98 dengue case patients, 5 (5%) were positive for both dengue NS1 antigen and antibody tests, 49 (50%) were positive only dengue NS1 antigen and 6 (6%) were positive only dengue antibody test. The combination of dengue NS1 antigen test and dengue antibody test had a sensitivity of 61% (60/98 dengue case patients) and a specificity of 91% (472/516 non-dengue control patients).

Using a positive cutoff value of 8 for *Burkholderia* antigen test, the test had a sensitivity of 27% (47/177 melioidosis case patients) and a specificity of 97% (424/437 non-melioidosis control patients). Using a positive cutoff value ranging from 7 to 36, Chikungunya antigen test, Zika antigen test, Chikungunya antibody test, Zika antibody test, *Leptospira* antibody test, *Orientia tsutsugamushi* antibody test, *Rickettsia typhi* antibody test gave a specificity of 95%.

Of all 414 cases with malaria, dengue or melioidosis, 236 (57%) had an accurate diagnosis made by DPP^®^ Fever Panel II Asia. All five cases with mixed infections had an inaccurate diagnosis made, including a positive result for pLDH but negative results for Dengue NS1 Ag and IgM Ab for a case with a mixed infection of malaria and dengue, and all negative test results for malaria, dengue and melioidosis for the remaining four cases. Of all 200 bacteraemia controls without malaria, dengue or melioidosis, 178 (89%) had accurate negative results for all malaria, dengue and melioidosis.

The results so far were derived using cutoff values targetting a specificity of at least 95%. A secondary analysis was conducted using cutoff values based on Youden’s index, implying the highest overall accuracy while affording equal weight to sensitivity and specificity (Table S2). Overall, this implied higher sensitivity and lower specificity, with the largest difference being in the dengue tests, where use of the Youden index cutoff would result in an increased sensitivity for the dengue IgM test to 66% but a corresponding decrease in specificity to 59%. For the combination of NS1 and IgM the sensitivity would increase to 88% (86/98 dengue case patients) while specificity would decrease to 56% (290/516 non-dengue control patients).

For the association between antigen levels and mortality, we observed an odds ratio of 2.34 (95% CI 1.16-4.72, p=0.02) for the association between the positivity of DPP^®^ CPS (CPS≥8) and mortality among melioidosis patients.

## Discussion

This study of patients hospitalized within 24 hours with community-acquired infection and sepsis at a referral hospital in northeast Thailand demonstrates that the DPP^®^ Fever Panel II Asia could be used to diagnose malaria, dengue and melioidosis with a performance that although considerably lower than reference tests, is comparable to other established RDTs for these tropical diseases. A key advantage of the DPP^®^ Fever Panel II Asia is its ability to support the diagnosis of several diseases in the array simultaneously with a low volume capillary sample, as compared to using several RDTs for each specific disease separately. Other potential, albeit speculative advantages of quantitative RDTs are their use to aid in diagnosis or prognosis of severe disease and adaptable cutoff thresholds for different epidemiological settings and clinical or public health requirements.

The DPP^®^ Fever Panel II is not yet available commercially and cutoff values that will determine the sensitivity and specificity have not been finalized. Here we used preliminary cutoffs that were calibrated to attain a high degree of specificity, which was considered a necessity if the tests are to offer added value over standard practice, which in many (but not all) settings is characterized by overuse of antimicrobial treatments. Even with this calibration for high specificity, the resulting sensitivity of pLDH and HRPII was comparable with findings from previous studies evaluating other leading malaria RDTs [20-23]. Among Pf malaria cases, the sensitivity of HRPII is higher than that of pLDH (94% vs. 73%), which is consistent with previous findings [22]. The sensitivity of pLDH among non-Pf malaria in our setting (79%) was comparable to that of CareStart® malaria pLDH (79%), but relatively lower than that of OptiMal-IT® pLDH (90%), previously evaluated in Southeast Asia [23].

The sensitivity and specificity of the DPP^®^ Dengue NS1 Antigen and IgM Antibody were comparable to the performance of other RDTs in previous studies [24-29]. As elsewhere, the accuracy of the DPP^®^ Dengue IgM test alone was poor (sensitivity of 11% with a corresponding specificity of 95%, or when using the Youden index sensitivity of 66% with a corresponding specificity of 59%). This is probably because most patients included in our study had a duration of symptoms prior to hospitalization of less than 5 days, and the test was performed on blood specimens collected within 24 hours of hospitalization; IgM antibodies are generally detectable in only about 50% of patients by days 3-5 after onset of symptoms [30].

The sensitivity of *B. pseudomallei* CPS Ag of DPP (27%) was comparable to the sensitivity of *B. pseudomallei* CPS detection of Active Melioidosis Detect® lateral flow immunoassay developed by InBios (31%) for the diagnosis of melioidosis [31]. The limited performance of antigen tests in unamplified blood is well described and low sensitivity compared with blood culture is to be expected [10]. Despite the low sensitivity, we found a clear association between CPS positivity and mortality among melioidosis patients, a finding that is consistent with a previous study [17] and could suggest that the use of CPS tests (in areas where better diagnostics are unavailable) is of high clinical utility. A similar association between antigen levels and severity is well demonstrated for HRPII in malaria (albeit this association might be stronger in plasma less than in whole blood) [16], and in some studies has been found for NS1 in dengue [18], although the literature on this association is mixed. Given the low number of deaths in the malaria and dengue cases we were unable to explore this association in our study.

One of the strengths of this study is the inclusion of cases and controls drawn from a large prospective observational study of patients presenting with community-acquired infection and sepsis in northeast Thailand, representing a real-world setting [8]. Additionally, all serum samples were drawn within 24 hours of admission to the study hospital which is ideal for evaluating point-of-care diagnostic tests.

A limitation of this study is that positive predictive and negative predictive values could not be estimated because of the case-control study design. Quantitative PCR assays for malaria and dengue were not performed. The cutoff values selected were to a great extent arbitrary. For most tests there will seldom be a single optimal threshold, whether due to heterogeneity in background exposure and immunity, or the different health and economic implications for patients with an incorrect test result. Our cutoff values corresponding with a specificity of at least 95% will certainly not be appropriate in all settings. Many diagnostic tests for malaria, dengue and melioidosis that reported higher sensitivity or higher specificity have used similarly arbitrary cutoffs, or in some instances Youden’s Index to determine the cutoff, which we also provide for comparability. The main disadvantage of Youden’s index is indifference between the sensitivity and specificity of the test [32]. In our study use of the Youden index mostly resulted in higher sensitivity but lower specificity, which in clinical settings would result in high rates of false positivity, misdiagnosis; in widely used RDTs for malaria and dengue potential this can imply under-treatment of bacterial infections [12-15]. We could not evaluate sensitivity and specificities of other pathogens the DPP^®^ Fever Panel II Asia is designed to test for, including Chikungunya infection, Zika infection, leptospirosis, scrub typhus and murine typhus as there were insufficient numbers of patients with these infections in the cohort. Other work is currently underway to evaluate the performance of the assay in detecting these infections. Further evaluation of the DPP^®^ Fever Panel II Asia with different settings and in the real clinical settings are needed.

In conclusion, the DPP^®^ Fever Panel II Asia offers the potential for rapid, simultaneous point of care testing for both antigen and antibodies for some of the key causes of febrile illness in Southeast Asia. We found the performance of the panels for the diagnosis of malaria, dengue and melioidosis to be similar to other established rapid tests for these pathogens, although as with other tests when compared with reference tests the accuracy for dengue was modest and the sensitivity for melioidosis was low. Notwithstanding these limitations and the need for further evaluations for its performance in diagnosing other pathogens on the panel, the DPP^®^ Fever Panel II Asia could substantially improve the management of febrile illness in much of rural Southeast Asia where access to laboratory testing is often unavailable.

## Supplementary Material

Table S1, Table S2

## Figures and Tables

**Table 1:** Sensitivity and specificity of DPP^®^ Fever Panel II Asia Antigen System and DPP^®^ Fever Panel II Asia IgM System among patients with malaria, dengue, melioidosis and bacteraemia

Panels	Cutoff values	Sensitivity	Specificity
**Antigen (Ag)**			
pLDH malaria Ag test	≥ 19	76% (108/143 malaria cases)	99% (464/471 non-malaria controls) ^a^
HRPII malaria Ag test	≥ 9	94% (75/80 Pf malaria cases)	98% (524/534 non-Pf malaria controls) ^b^
Dengue NS1 Ag test	≥ 22	55% (54/98 dengue cases)	95% (491/516 non-dengue controls) ^c^
*B. pseudomallei* CPS Ag test	≥ 8	27% (47/177 melioidosis cases)	97% (424/437 non-melioidosis controls) ^d^
Chikungunya Ag test	≥ 12	Not applicable	95% (586/614 patients) ^e^
Zika NS1 Ag test	≥ 11	Not applicable	94% (580/614 patients) ^e^
**Antibody (Ab)**			
Dengue Ab test	≥ 56	11% (11/98 dengue cases)	95% (491/516 non-dengue controls) ^c^
Chikungunya Ab test	≥ 21	Not applicable	95% (585/614 patients) ^e^
Zika Ab test	≥ 18	Not applicable	95% (585/614 patients) ^e^
*Leptospira* Ab test	≥ 36	Not applicable	95% (585/614 patients) ^e^
*O. tsutsugamushi* Ab test	≥ 7	Not applicable	97% (595/614 patients) ^e^
*R. typhi* Ab test	≥ 10	Not applicable	95% (584/614 patients) ^e^
**Used as a combination**			
pLDH + HRPII malaria Ag test	≥ 19, ≥ 9	91% (130/143 malaria cases)	97% (458/471 non-malaria controls) ^a^
Dengue NS1 Ag + Ab test	≥ 22, ≥ 56	61% (60/98 dengue cases)	91% (472/516 non-dengue controls) ^c^

Patients with bacteraemia included patients with blood culture positive for *E. coli*, *K. pneumoniae* and *S. aureus*

a included patients with bacteraemia, dengue and melioidosis

b included patients with bacteraemia, *Plasmodium vivax* malaria, dengue and melioidosis

c included patients with bacteraemia, malaria and melioidosis

d included patients with bacteraemia, malaria and dengue

e included patients with bacteraemia, malaria, dengue and melioidosis.
