# Supplementary material for: Sensitivity and specificity of DPP® Fever Panel II Asia in the diagnosis of malaria, dengue and melioidosis
Source: J Med Microbiol. Author manuscript; Available in PMC 2022 Oct 13. (PMC7613707; doi:10.1099/jmm.0.001584)
Supplement: Table S1, Table S2 [file EMS153102-supplement-Table_S1__Table_S2.docx]

**Table S1**. **Baseline characteristics of patients with malaria, dengue, melioidosis and bacteraemia included in the analysis**

| **Characteristics** | **Malaria***  **(n=143)** | **Dengue***  **(n=98)** | **Melioidosis***  **(n=177)** | **Bacteraemia**  **(n=200)** |
| --- | --- | --- | --- | --- |
| Male gender (n [%]) | 128 (90%) | 43 (44%) | 123 (69%) | 77 (39%) |
| Age (years)  (median [IQR, range]) | 39  (30-50, 19-76) | 27  (22-37, 18-75) | 55  (46-65, 20-89) | 64  (55-74, 18-94) |
| Duration of symptoms |  |  |  |  |
| ≤ 2 days | 19 (13%) | 22 (22%) | 40 (23%) | 123 (62%) |
| 3-4 days | 73 (51%) | 46 (47%) | 45 (25%) | 44 (22%) |
| 5-7 days | 41 (29%) | 26 (27%) | 48 (27%) | 21 (11%) |
| > 7 days | 10 (7%) | 4 (4%) | 44 (25%) | 12 (6%) |
| 28-day mortality (n [%]) | 1 (0.7%) | 4 (4%) | 94 (53%) | 36 (18%) |

**Footnote of Supplementary** **1**: *The three patients with mixed infection between melioidosis and dengue are shown in both groups; the patient with mixed infection between malaria and dengue is shown in both groups; and the patient with mixed infection between melioidosis and *K. pneumoniae* bacteraemia is shown only in the melioidosis group.

**Table S2. Sensitivity and specificity of DPP® Fever Panel II Asia Antigen System and DPP® Fever Panel II Asia IgM System among patients with malaria, dengue, melioidosis and bacteraemia, using cutoff values defined by Youden’s index**

| **Panels** | **Cut-off values** | **Sensitivity** | **Specificity** |
| --- | --- | --- | --- |
| **Antigen (Ag)** |  |  |  |
| pLDH malaria Ag test | ≥ 19 | 76% (108/143 malaria cases) | 99% (464/471 non-malaria controls) ^a^ |
| HRPII malaria Ag test | ≥ 19 | 94% (75/80 Pf malaria cases) | 99% (533/534 non-Pf malaria controls) ^b^ |
| Dengue NS1 Ag test | ≥ 16 | 66% (65/98 dengue cases) | 92% (473/516 non-dengue controls) ^c^ |
| *B. pseudomallei* CPS Ag test | ≥ 7 | 29% (52/177 melioidosis cases) | 94% (412/437 non-melioidosis controls) ^d^ |
| **Antibody (Ab)** |  |  |  |
| Dengue Ab test | ≥ 7 | 66% (65/98 dengue cases) | 59% (305/516 non-dengue controls) ^c^ |
| **Used as a combination** |  |  |  |
| pLDH + HRPII malaria Ag test | ≥ 19, ≥ 19 | 90% (129/143 malaria cases) | 99% (464/471 non-malaria controls) ^a^ |
| Dengue NS1 Ag + Ab test | ≥ 16, ≥ 7 | 88% (86/98 dengue cases) | 56% (290/516 non-dengue controls) ^c^ |

**Footnote of Table S2**. Patients with bacteraemia included patients with blood culture positive for *E. coli*, *K. pneumoniae* and *S. aureus* ^a^ included patients with bacteraemia, dengue and melioidosis ^b^ included patients with bacteraemia, *Plasmodium vivax* malaria, dengue and melioidosis ^c^ included patients with bacteraemia, malaria and melioidosis ^d^ included patients with bacteraemia, malaria and dengue.
